# Supplementary material for: Rapid Identification of Pollen- and Anther-Specific Genes in Response to High-Temperature Stress Based on Transcriptome Profiling Analysis in Cotton
Source: Int J Mol Sci. 2022 Mar 21;23(6):3378. doi: 10.3390/ijms23063378 (PMC8954629; doi:10.3390/ijms23063378)
Supplement: Supplementary file 1 [file ijms-23-03378-s001.zip › Table S/Table S14 Primer used in this study.pdf]

Table S13. Primers used for RT and qRT-PCR in this study.

Gene ID	Primers	Sequence(5′-3′)
GhUBIQUITIN7	F	GAAGGCATTCCACCTGACCAAC
	R	CTTGACCTTCTTCTTCTTGTGCTTG
Ghir_D12G012350	F	GCAATGGCGGAAGTAATAAAGC
	R	ATGTAAAAGCCGTTGATGGTCG
Ghir_A05G027730	F	ACGCCTCCATCTGAAACCTGT
	R	ATAGTTGACAGCAGAATCAGCCC
Ghir_D11G001020	F	TGACTGCTGTAACGCACTCCAAT
	R	CACCACCATTGCTTATTACCCATT
Ghir_A05G021300	F	ACAGGGACATGAAGAACTACAAGG
	R	GAAACCTTTAATGGGTCCTCGTC
Ghir_D12G027340	F	CCTCAATAGGTGGTTCAGCAGTC
	R	GGTAACAAGCCCCATTAAGCA
Ghir_D12G001210	F	CCTTGCCTTACTTAAAGGGGG
	R	GAACAGGGAGTGATGGATAACGAG
Ghir_A12G023790	F	TTTCAACTCTTCGCCTGGGACT
	R	AGACTTCAAATTCTGGACCACTCAA
Ghir_D12G020020	F	CTCCATGAACCGCAACAGTC
	R	CGACAACTCCCGTATTATTCCG
Ghir_D05G009200	F	CACAGTTGGCATTCTGGTAGCA
	R	TTTTGAACATCGTCTACACCCCT
Ghir_D05G004060	F	ATGGGTCGTGGTCTGGGAT
	R	AAGGCAGCCGAGATAACCG
Ghir_A06G007780	F	CTGAAGGGCATAGTAAACTCGGT
	R	TTGTTAGCCTCATCGGTAAAGCA
Ghir_D11G016190	F	GATACCCCCGAAGATGAAGACG
	R	ACCCTTCCAAATCCAAACACG
Ghir_A05G036740	F	AATGACACAATCTTCGGCGG
	R	CAAAGCACACAAAGCCCAAAC
Ghir_D11G007200	F	AGCAGTTCCCCTCCTACACCA
	R	CTGTTCACACATTCTATGCCGC
Ghir_D05G031690	F	GAAAGCGGAAATGCGAAACTC
	R	TTGACCCTCCTCCACTCGTTA

Ghir_A02G005650	F	TCACAAACGGATGGGAACTCA
	R	GCCAAAGGGTCCTCAGCATAG
