# Supplementary figures and images for: Rapid Identification of Pollen- and Anther-Specific Genes in Response to High-Temperature Stress Based on Transcriptome Profiling Analysis in Cotton
Source: Int J Mol Sci. 2022 Mar 21;23(6):3378. doi: 10.3390/ijms23063378 (PMC8954629; doi:10.3390/ijms23063378)

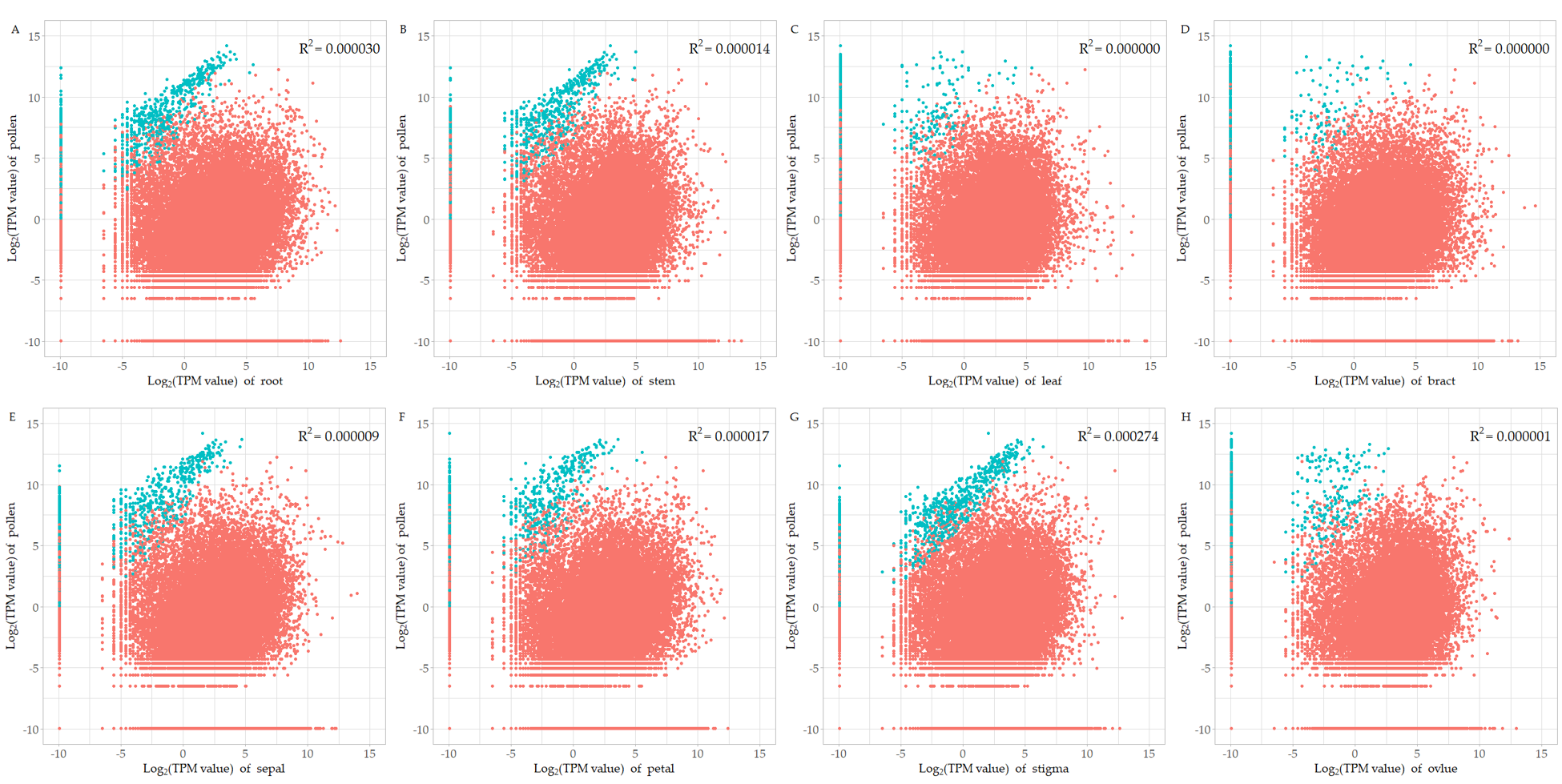

Supplement: Supplementary file 1 [file ijms-23-03378-s001.zip › Fig S/Figure S1.png]

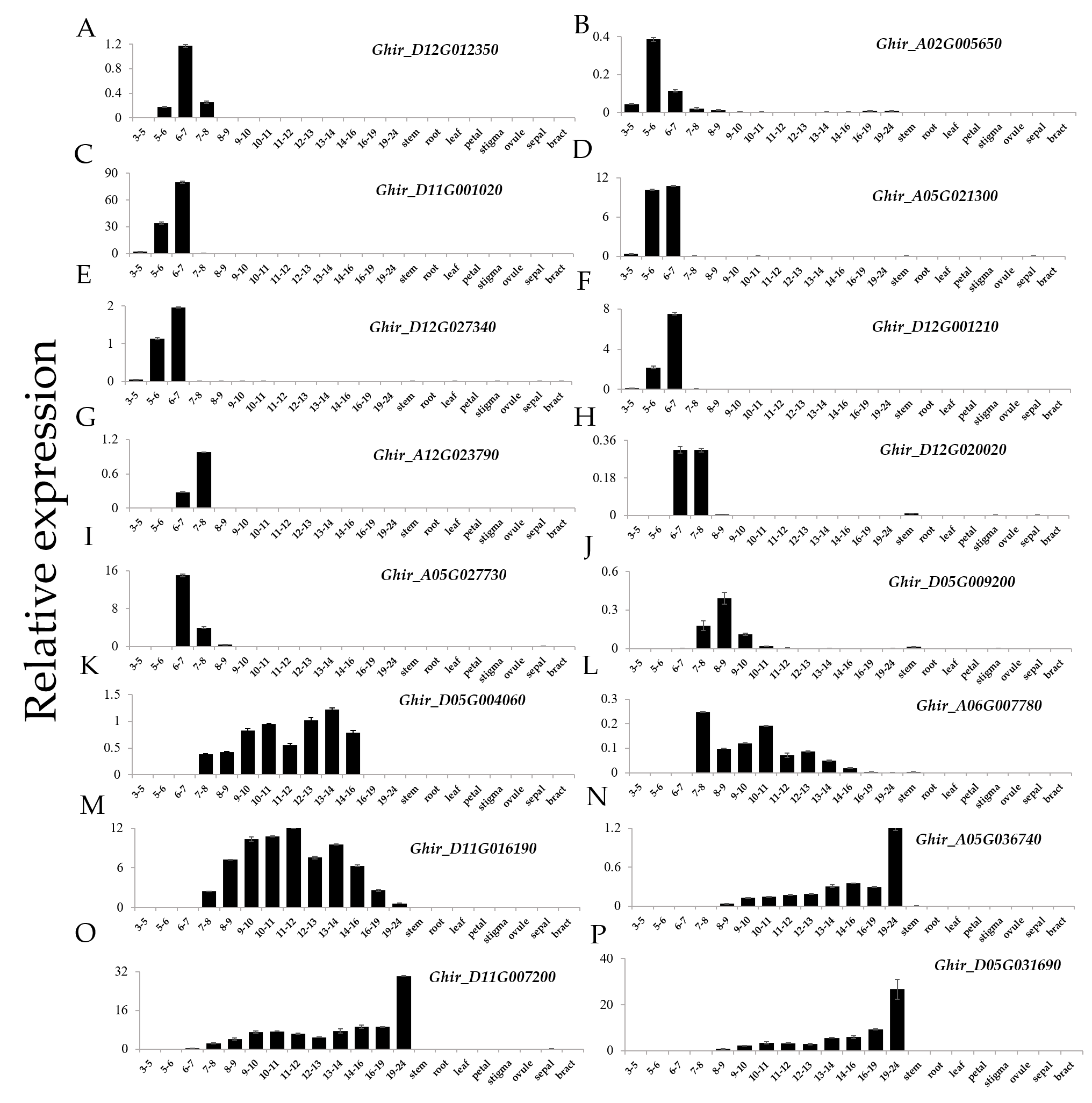

Supplement: Supplementary file 1 [file ijms-23-03378-s001.zip › Fig S/Figure S2.png]

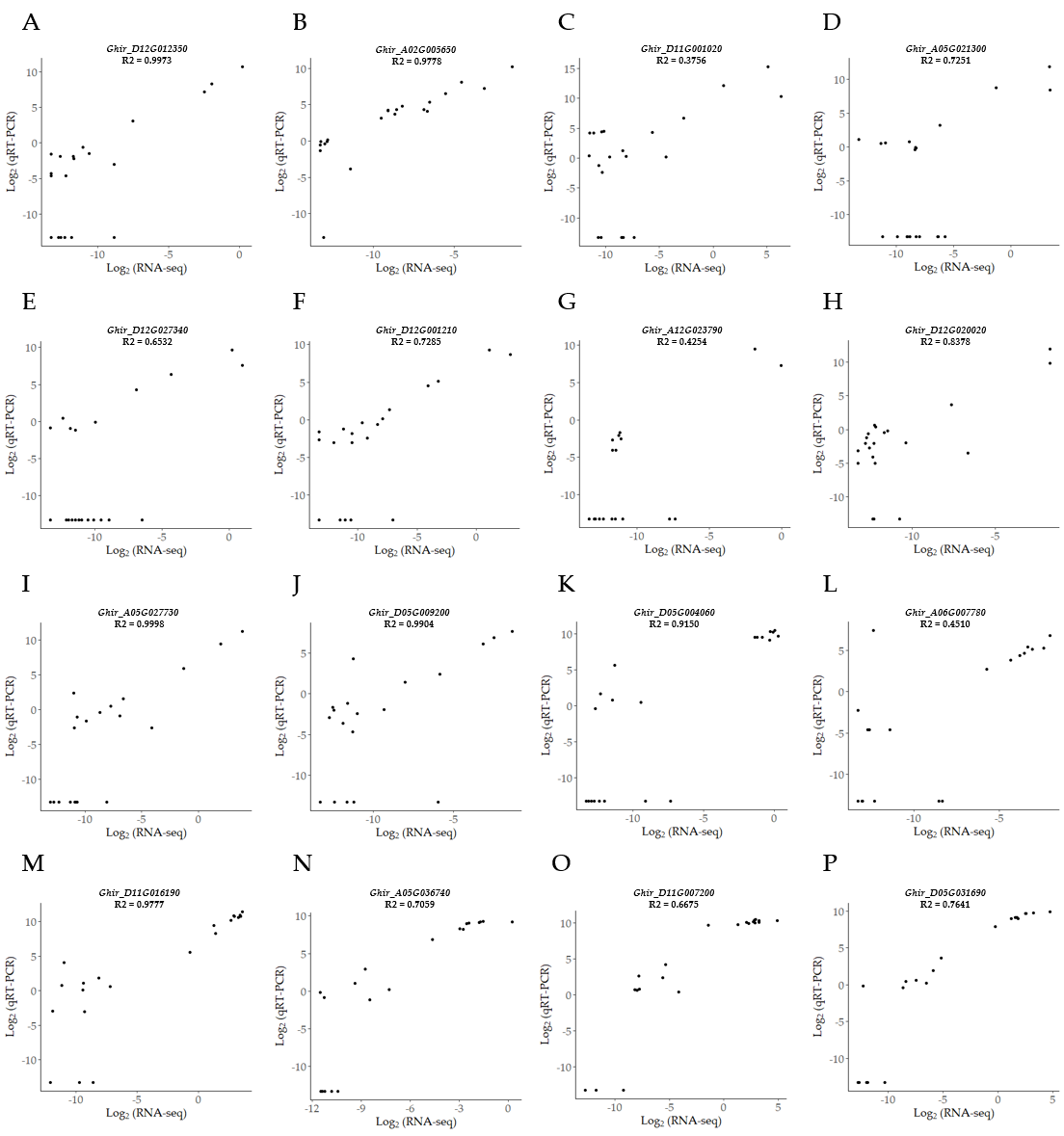

Supplement: Supplementary file 1 [file ijms-23-03378-s001.zip › Fig S/Figure S3.png]

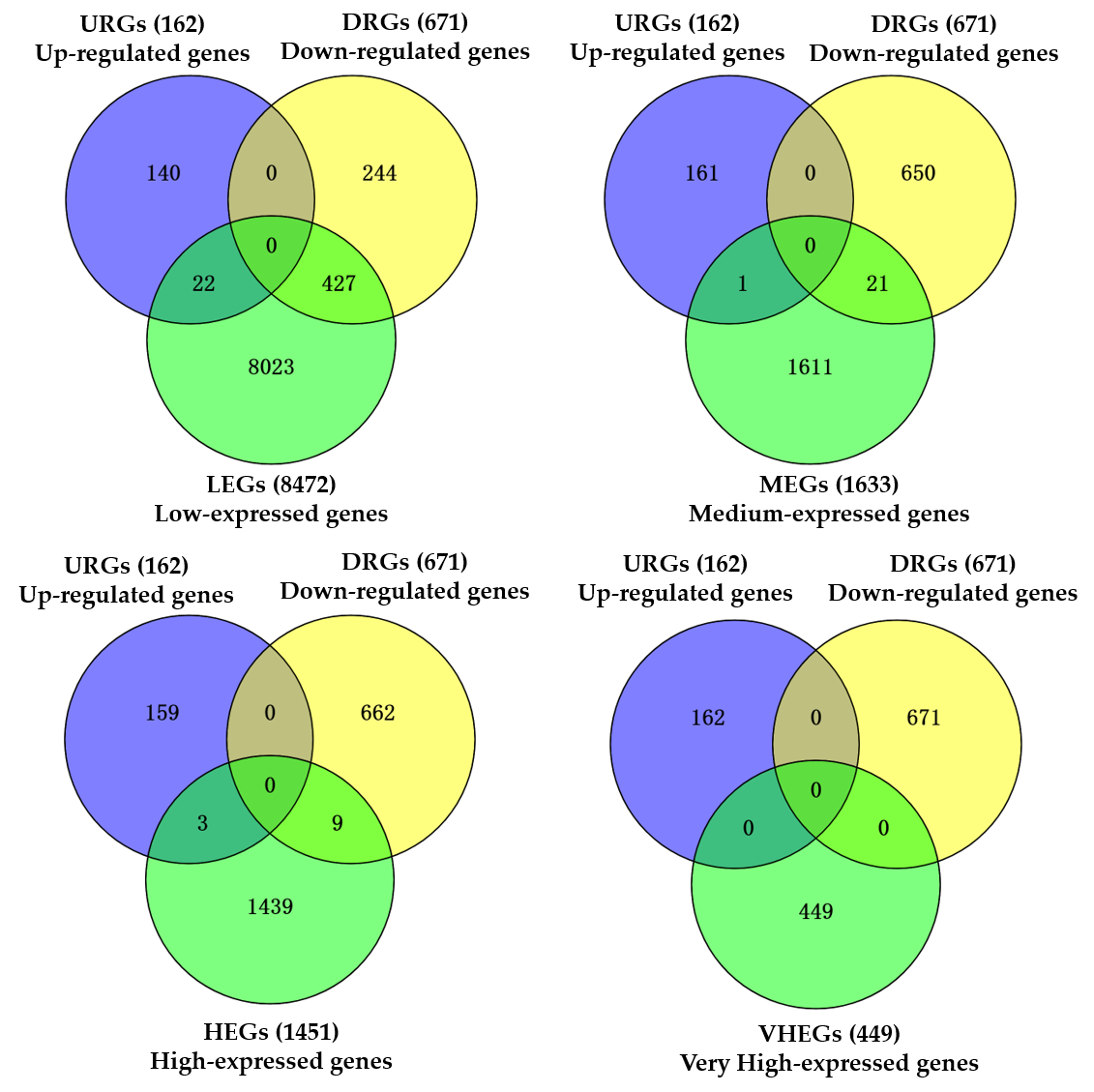

Supplement: Supplementary file 1 [file ijms-23-03378-s001.zip › Fig S/Figure S4.png]
